# pyComBat, a Python tool for batch effects correction in high-throughput molecular data using empirical Bayes methods

**DOI:** 10.1186/s12859-023-05578-5

**Published:** 2023-12-07

**Authors:** Abdelkader Behdenna, Maximilien Colange, Julien Haziza, Aryo Gema, Guillaume Appé, Chloé-Agathe Azencott, Akpéli Nordor

**Affiliations:** 1 Epigene Labs, Paris, France; 2 MINES ParisTech, CBIO-Centre for Computational Biology, PSL Research University, 75006 Paris, France; 3 Institut Curie, PSL Research University, 75005 Paris, France; 4 INSERM, U900, 75005 Paris, France; 5 University of Edinburgh, Edinburgh, UK

**Keywords:** Batch effects, Transcriptomics, Bayesian statistics, Open source

## Abstract

**Background:**

Variability in datasets is not only the product of biological processes: they are also the product of technical biases. ComBat and ComBat-Seq are among the most widely used tools for correcting those technical biases, called batch effects, in, respectively, microarray and RNA-Seq expression data.

**Results:**

In this technical note, we present a new Python implementation of ComBat and ComBat-Seq. While the mathematical framework is strictly the same, we show here that our implementations: (i) have similar results in terms of batch effects correction; (ii) are as fast or faster than the original implementations in R and; (iii) offer new tools for the bioinformatics community to participate in its development. pyComBat is implemented in the Python language and is distributed under GPL-3.0 (https://www.gnu.org/licenses/gpl-3.0.en.html) license as a module of the inmoose package. Source code is available at https://github.com/epigenelabs/inmoose and Python package at https://pypi.org/project/inmoose.

**Conclusions:**

We present a new Python implementation of state-of-the-art tools ComBat and ComBat-Seq for the correction of batch effects in microarray and RNA-Seq data. This new implementation, based on the same mathematical frameworks as ComBat and ComBat-Seq, offers similar power for batch effect correction, at reduced computational cost.

## Background

Batch effects are the product of technical biases, such as variations in the experimental design or even atmospheric conditions [1, 2]. They particularly reveal themselves when merging different datasets, which have likely been built under different conditions. If not corrected, these batch effects may lead to incorrect biological insight, since the variability can be wrongly interpreted as the product of a biological process.

Multiple methods exist that address this problem. They include approaches related to frequentist statistics, such as simple normalization [3, 4] or principal component analysis [5]; and machine learning, such as support-vector machines [6]. One of their main flaws is, however, their incapacity to handle low sample sizes or more than two batches at the same time [7].

ComBat, originally implemented in the R library sva [8], is based on the mathematical framework defined in [9]. This tool leverages a parametric and non-parametric empirical Bayes approach for correcting the batch effect in microarray datasets that works for small sample sizes or in the presence of outliers. This approach is based on the fact that microarray expression data are generally distributed according to a log-normal distribution [10]. Note that the parametric method requires strong assumptions but is largely faster than the non-parametric approach.

ComBat-Seq, also implemented in the R library sva, is based on a similar mathematical framework, where normal distributions are replaced by negative binomial distributions, to better reflect the statistical behavior of RNA-Seq raw counts data [11].

We recall the details of the mathematical models of ComBat and ComBat-Seq in Table 1.

**Table 1:** Details of the mathematical models of ComBat and ComBat-Seq

ComBat model	ComBat-Seq model
${y}_{gij} \sim Normal({\mu }_{gi}; {\phi }_{gi})$	${y}_{gij} \sim NegativeBinomial({\mu }_{gij}; {\phi }_{gi})$
${\mu }_{gi} = {\alpha }_{g} + {X}_{j} {\beta }_{g} + {\gamma }_{gi}$	$log {\mu }_{gij} = {\alpha }_{g} + {X}_{j} {\beta }_{g} + {\gamma }_{gi}$
$Var({y}_{gij}) = {\phi }_{gi}$	$Var({y}_{gij}) = {\mu }_{gij} + {\phi }_{gi} \mu {}_{gij}^{2}$

${y}_{gij}$ denotes the expression value for gene $g$ in sample $j$ from batch $i$, ${\alpha }_{g}$ denotes a gene-wise baseline expression level, ${X}_{j}$ is a vector of covariates for sample $j$, ${\beta }_{g}$ is the vector of corresponding regression coefficients for gene $g$, ${\gamma }_{gi}$ is the additive (impacting the mean) batch effect and ${\phi }_{gi}$ is the multiplicative (impacting the variance) batch effect

We introduce in this article pyComBat, a new Python tool implementing ComBat (function pycombat_norm) and ComBat-Seq (function pycombat_seq), following the same mathematical frameworks. Note that the term “pyComBat” implicitly refers to the function pycombat_norm (resp. pycombat_seq) when compared to ComBat (resp. ComBat-Seq). In comparison to both the R implementation and the existing Python implementation of ComBat in the single-cell analysis library Scanpy [12], we show that pyComBat yields similar results for adjusting for batch effects in microarray data, but is generally faster, in particular for the usually slow, but more loose, non-parametric method. Similarly, in comparison to the R implementation, we show that pyComBat yields identical results for adjusting for batch effects in RNA-Seq data, and is generally faster. To our knowledge, it is the sole Python implementation of ComBat-Seq.

## Implementation of pyComBat

pyComBat is a Python 3 implementation of ComBat and ComBat-Seq. It mostly uses generic libraries like Pandas [13] or NumPy [14] to mimic ComBat and ComBat-Seq, following the exact same mathematical framework.

Two important features are not directly related to the performance of the software but are of utmost importance. First, pyComBat is available as an open-source software under a GPL-3.0 license, which means anyone can use, modify, distribute and share it. Opening pyComBat to the bioinformatics Python community is the best way for maintaining and improving it, while increasing its robustness. Second, the reliability of pyComBat has been thoroughly checked, using a bench of unit tests (code coverage measured at 88% with Python module “coverage”) serving both as functional tests (to ensure the proper functioning of each submodule) and as non-regression tests (to ease maintenance).

## Results: comparing pyComBat with ComBat and ComBat-Seq

### Datasets used and preprocessing

For software validation, we created two microarray and two RNA-Seq meta-datasets from public data: one on Ovarian Cancer (6 microarray datasets), one on Multiple Myeloma (4 microarray datasets), one on Breast Cancer (originally used for the validation of ComBat-Seq [11], 2 RNA-Seq datasets) and one on Colon Cancer (2 RNA-Seq datasets) All meta-datasets are described in more detail in Table 2.

**Table 2:** Composition of each meta-dataset used for benchmarking pyComBat, Scanpy’s implementation of ComBat, ComBat and ComBat-Seq

Dataset	Reference(s)
*Ovarian Cancer (microarray)*	
GSE18520	[22]
GSE66957	
GSE69428	[23]
GSE9891	[24]
GSE26712	[25, 26]
GSE38666	[27, 28]
*Multiple Myeloma (microarray)*	
GSE5900	[29–31]
GSE66291	[32, 33]
GSE68891	
GSE122231	[34, 35]
*Breast cancer (RNA-Seq)*	
GSE83083	[36]
GSE59765	[37]
*Colon cancer (RNA-Seq)*	
phs000892.v6.p1	[38]
phs000178.v11.p8	[39]

Microarray data were normalized with the rma function from the affy R package (v.1.68.0) [15] which applies a log2 transformation and ensures the normal distribution of normalized data, while the raw counts were directly acquired for RNA-Seq data as suggested in the ComBat-seq documentation [11].

We then compared

- ComBat, Scanpy’s implementation of ComBat and pyComBat on the microarray datasets on one hand,
- ComBat-Seq and pyComBat on the RNA-Seq datasets.

for (*i*) efficacy for batch effect correction and (*ii*) computation time.

### Batch effect correction

**Fig. 1:**
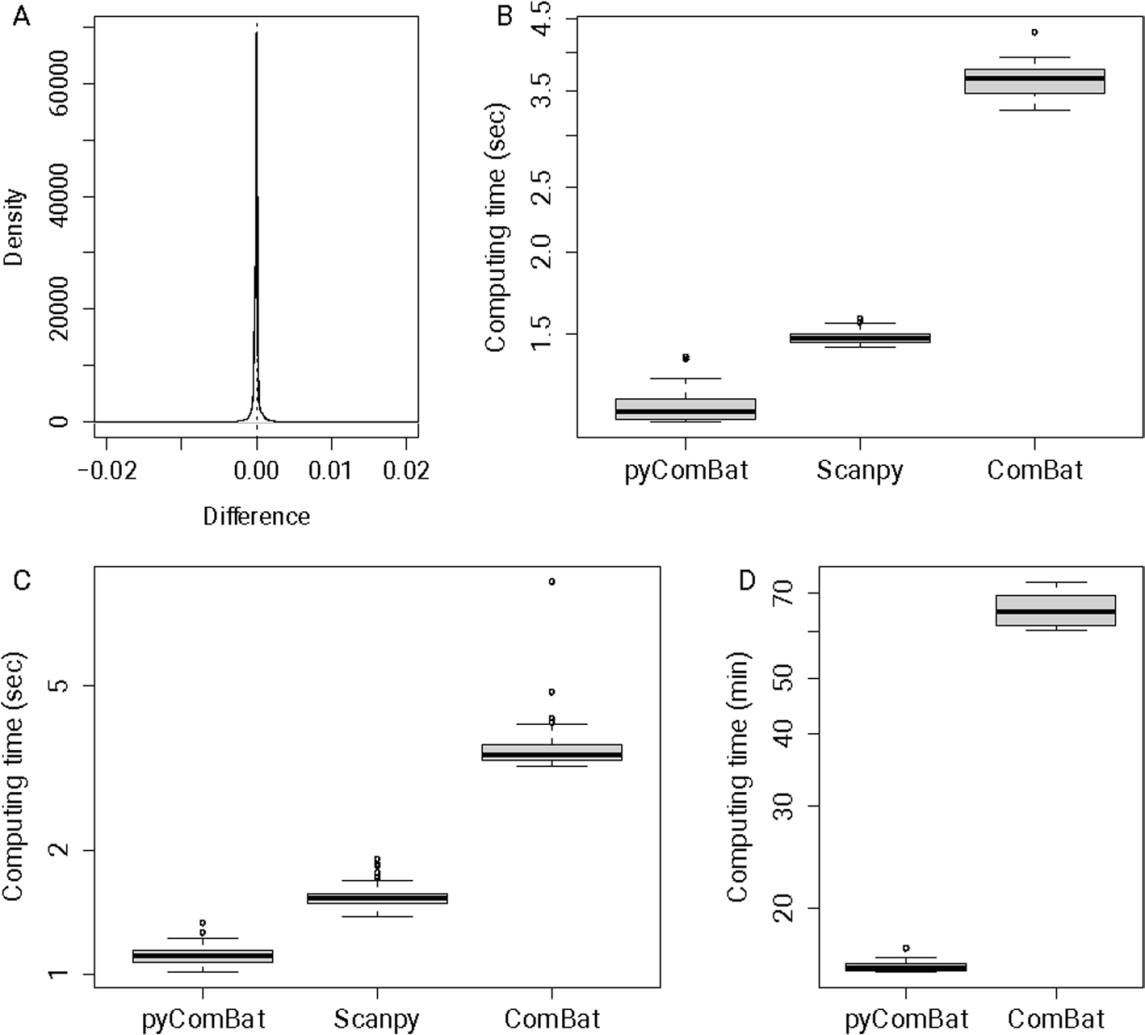
Performance of pyComBat *vs*. Combat *vs.* Scanpy’s implementation of ComBat. **A** Distribution of the relative differences between the expression matrices corrected for batch effects, respectively by ComBat and pyComBat (parametric version), on the Ovarian Cancer dataset. The vertical dotted line corresponds to zero. **B** Computation time in seconds for pyComBat, Scanpy and ComBat for the parametric method, on the Multiple Myeloma dataset. The y-axis is in a log scale. **C** Computation time in seconds for pyComBat, Scanpy and ComBat for the parametric method, on the Ovarian Cancer dataset. The y-axis is in a log scale. **D** Computation time in minutes for pyComBat (left) and ComBat (right) for the non-parametric method, on the Ovarian Cancer dataset. The y-axis is in a log scale

As an implementation of the ComBat and ComBat-Seq algorithms, pyComBat is expected to have similar, if not identical, power in terms of batch effects correction. This is confirmed in Fig. 1A, which shows the distribution of relative differences between the outputs of ComBat and pyComBat, on the Ovarian Cancer dataset (mean = − 1.06 × 10^–7^, 95% CI = [− 1.28 × 10^–3^, 1.32 × 10^–4^]). As expected, the differences are distributed closely around zero with a relative squared error of 1.7 × 10^–7^, suggesting that the variability relative to the use of ComBat or pyComBat is negligible compared to the intrinsic variability of the data.. The slight variability can be explained by the difference between optimization routines in R and Python (Numpy): while small differences in the fitted distribution parameters have little to no impact for the vast majority of data points, they may be amplified by data adjustment for data points located far away in the distribution tails.

Additionally, both ComBat-Seq and pyComBat produce the exact same output on the Breast Cancer and Colon Cancer datasets. Despite the aforementioned differences in optimization routines between R and Python, the slight variations in the fitted distribution parameters are leveled out by rounding to integers during data adjustment as both tools output adjusted integer counts. Given the long-tailed nature of the negative binomial distribution, one would expect that even slight variations in parameter values can impact data points far in the distribution tail, a phenomenon completely absent in our experiments.

To sum up, it is highly unlikely to obtain such similar results unless pyComBat implements the same algorithms as ComBat and ComBat-Seq.

### Computation time

Computation time is evaluated by running pyComBat (resp. Scanpy’s implementation of ComBat and ComBat itself) respectively 100 times on both microarray datasets presented in Section “Results: comparing pyComBat with ComBat and ComBat-Seq”, with the parametric approach. As Scanpy doesn’t handle the non-parametric approach, only ComBat and pycombat_norm have been tested with it, on the Ovarian Cancer dataset. As for pycombat_seq and ComBat-Seq, they have been run respectively 50 times on the Breast Cancer dataset and 20 times on the Colon Cancer dataset. The reduced number of runs on RNA-Seq datasets is due to the longer, and less variable, computation times.

Owing to Python (Numpy) efficiency in handling matrix operations and matrix manipulations as well as thorough optimization of our code, pyComBat is as fast or even faster than ComBat. The parametric version of the pyComBat performs 4–5 times as fast as ComBat, and around 1.5 times as fast as the Scanpy implementation of ComBat, in terms of computation time (Fig. 1B, C), on both datasets.

Similar results are observed with the non-parametric version (Fig. 1D), which is inherently more time consuming, but also less dependent on the distribution of the data. In this case, pyComBat is also approximately 4–5 times faster than ComBat, going from more than an hour to around 15 min.

**Fig. 2:**
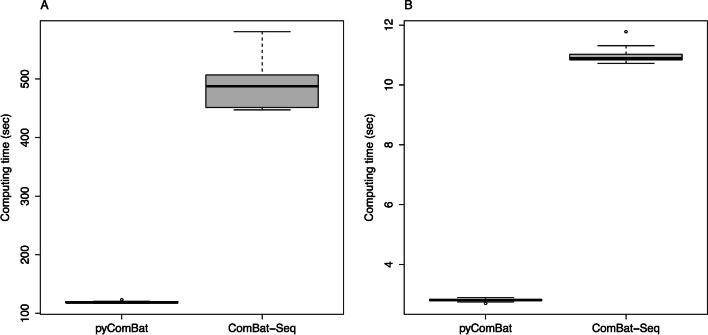
Performance of pyComBat *vs.* ComBat-Seq. **A** Computation time in seconds for pyComBat and ComBat-Seq, on the Colon Cancer dataset. **B** Computation time in seconds for pyComBat and ComBat-Seq, on the Breast Cancer dataset

Finally, pyComBat appears to be 4–5 times faster than ComBat-Seq on both RNA-Seq datasets (Fig. 2A, B).

### Downstream analysis

The main goal of batch effect correction in transcriptomic is to produce unbiased and more powerful downstream analysis. We show here that the slight differences in corrected gene expression between ComBat and pyComBat have little to no impact on the output of differential gene expression analysis. Such an analysis is not needed on our RNA-Seq datasets, as ComBat-Seq and pyComBat output the exact same adjusted count matrix.

**Fig. 3:**
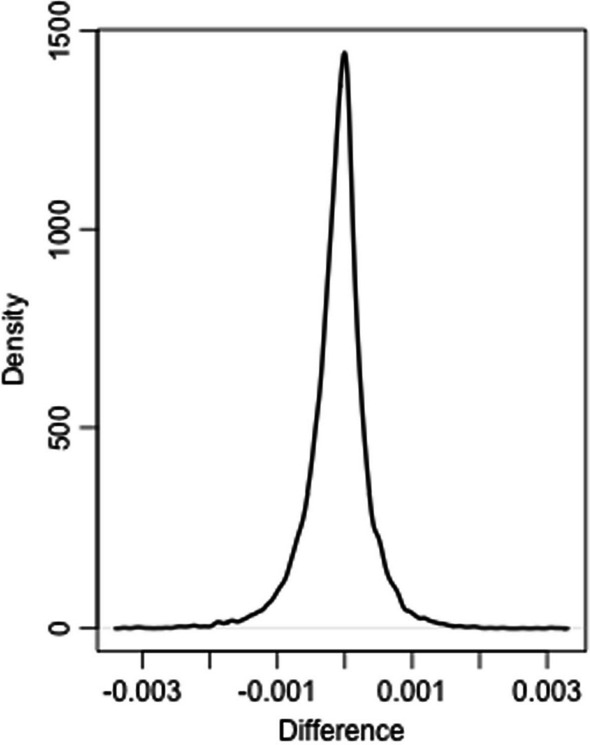
Differences of logFoldChange between ComBat and pyComBat corrected data for the Ovarian microarray dataset, for a differential expression analysis between primary tumor and normal tissue samples using Limma

We have applied differential gene expression analysis on the Ovarian Cancer dataset, to compare groups based on sample types: Primary tumors against Normal tissues. Both ComBat and pyComBat corrected data have been analyzed using the Limma R package (v3.56.2) [16], comparing 62 Normal tissue and 615 Primary tumor samples. As shown in Fig. 3, the differences in batch effects corrections have a small impact on the logFoldChange differences (mean = − 1.02·× 10^–4^, 95% CI = [− 1.15·× 10^–3^, 7.88·×·10^–4^]) between both differential analyses. Moreover, with usual thresholds found in the literature (*i.e.* logFoldChange > 1.5 and fdr < 0.05), the selected genes are the same, which suggests that ComBat and pyCombat can be used interchangeably before downstream analyses.

## Discussion and conclusion

We present pyComBat, a new Python implementation of ComBat and ComBat-Seq, the most commonly used software for batch effects correction on high-throughput molecular data. Our implementation offers the same correcting power, with shorter computation time for the parametric method compared to other implementations, and significantly shorter time for the time-consuming non-parametric version compared to the original R implementations. This reduced computing time opens perspectives for a more generic use of the non-parametric approach to a larger range of datasets.

As ComBat and pyComBat (pycombat_norm) assume the normal distribution of the input data, data processing should comply with this assumption. Microarray raw data generally follow a log-normal distribution, and R’s rma function applies a log-transformation after the normalization step, which ensures the normal distribution of the input data. However, other normalization tools, *e.g.* MAS5, do not log-transform the data. It is up to the user to check the distribution of their data and eventually apply a transformation accordingly. ComBat-seq and pyComBat (pycombat_seq) both work on data that follow a negative binomial distribution, which is the distribution of raw counts in RNA-Seq data. No preprocessing of the raw counts is thus needed.

Despite the historical prevalence of R, Python is gaining momentum in the bioinformatics landscape. Python is a general-purpose programming language, widely used in fields related to bioinformatics: data science, machine learning and AI, orchestration, visualization, etc. The versatility and wide adoption of Python eases interoperability of bioinformatics tools with tools from other domains of expertise. We believe that this interdisciplinary capabilities, along with its general-purpose abilities, gives Python a substantial edge over R, targeted at statistical applications, to grow as the language of choice for bioinformatic tools. By contributing to a single-language unified ecosystem, we hope to eliminate the need to interface several languages (typically R and Python), a common source of technical difficulties and of computational inefficiency. Workflow languages (such as Snakemake [17] or Nextflow [18]) and interoperability libraries (such as rpy2 [19]) still need to translate and copy data from one language to the other. This is a source of inefficiency and of limitations—depending on the supported data formats—especially when the analysis goes back and forth between languages. We thus advocate the necessity to port reference tools from R to Python. Sign of this trend, state-of-the-art tools have been directly developed in Python (*e.g.* lifelines [20], a library dedicated to survival analysis, scanpy [12] (a library dedicated to the analysis of single-cell omic data) or quickly ported in Python from R (*e.g.* harmony [21], an R library for integrating single cell data, DESeq2, an R library for differential expression analysis).

Furthermore, porting a tool from one language to another provides an opportunity window to improve both functionality and performance. For instance, pyComBat improves over ComBat-Seq by supporting reference batches.

The recent advent of large language models (LLMs) with coding capabilities, such as Starcoder, GPT4 or GitHub Copilot, represent a great opportunity to accelerate this porting process. Yet, human intervention remains necessary:

- to ensure the correctness of the ported code. Beyond hallucinations, LLMs remain statistic-based prediction models, and may thus introduce errors in the implementation, at the risk of changing the mathematical logic;
- to take full advantage of the performance and functionality improvement opportunity window. For instance, ComBat-Seq code is a mix of R and C++, and porting it to Python required adapting the C++ code to interface with Python instead of R.

Note however that the work on pyComBat was undertaken before the wide diffusion of LLM-based coding tools.

We have attached importance to making the software open source, coupled with comprehensive documentation. We built a robust set of test cases, in an effort to encourage larger participation from the community. We believe that this will be benefiting the Python bioinformatics community and opening the way towards the translation of other widely used software from R to Python.

## Data Availability

The datasets analyzed during the current study are available in the GEO and dbGAP repositories:—https://www.ncbi.nlm.nih.gov/geo/query/acc.cgi?acc=GSE18520—https://www.ncbi.nlm.nih.gov/geo/query/acc.cgi?acc=GSE66957—https://www.ncbi.nlm.nih.gov/geo/query/acc.cgi?acc=GSE69428—https://www.ncbi.nlm.nih.gov/geo/query/acc.cgi?acc=GSE9891—https://www.ncbi.nlm.nih.gov/geo/query/acc.cgi?acc=GSE26712—https://www.ncbi.nlm.nih.gov/geo/query/acc.cgi?acc=GSE38666—https://www.ncbi.nlm.nih.gov/geo/query/acc.cgi?acc=GSE5900—https://www.ncbi.nlm.nih.gov/geo/query/acc.cgi?acc=GSE66291—https://www.ncbi.nlm.nih.gov/geo/query/acc.cgi?acc=GSE68891—https://www.ncbi.nlm.nih.gov/geo/query/acc.cgi?acc=GSE122231—https://www.ncbi.nlm.nih.gov/geo/query/acc.cgi?acc=GSE83083—https://www.ncbi.nlm.nih.gov/geo/query/acc.cgi?acc=GSE59765—https://www.ncbi.nlm.nih.gov/projects/gap/cgi-bin/study.cgi?study_id=phs000892.v6.p1—https://www.ncbi.nlm.nih.gov/projects/gap/cgi-bin/study.cgi?study_id=phs000178.v11.p8.

## Availability and requirements

Project name: InMoose. Project home page: https://github.com/epigenelabs/inmoose. Operating system: Platform independent. Programming language: Python. Other requirements: Numpy, Scipy License: GNU GPL3. Any restrictions to use by non-academics: None.
